# Conservation of the Human Integrin-Type Beta-Propeller Domain in Bacteria

**DOI:** 10.1371/journal.pone.0025069

**Published:** 2011-10-13

**Authors:** Bhanupratap Chouhan, Alexander Denesyuk, Jyrki Heino, Mark S. Johnson, Konstantin Denessiouk

**Affiliations:** 1 Department of Biosciences, Åbo Akademi University, Turku, Finland; 2 Turku Center for Biotechnology, University of Turku and Åbo Akademi University, Turku, Finland; 3 Department of Biochemistry and Food Chemistry, University of Turku, Turku, Finland; MRC National Institute for Medical Research, United Kingdom

## Abstract

Integrins are heterodimeric cell-surface receptors with key functions in cell-cell and cell-matrix adhesion. Integrin α and β subunits are present throughout the metazoans, but it is unclear whether the subunits predate the origin of multicellular organisms. Several component domains have been detected in bacteria, one of which, a specific 7-bladed β-propeller domain, is a unique feature of the integrin α subunits. Here, we describe a structure-derived motif, which incorporates key features of each blade from the X-ray structures of human αIIbβ3 and αVβ3, includes elements of the FG-GAP/Cage and Ca^2+^-binding motifs, and is specific only for the metazoan integrin domains. Separately, we searched for the metazoan integrin type β-propeller domains among all available sequences from bacteria and unicellular eukaryotic organisms, which must incorporate seven repeats, corresponding to the seven blades of the β-propeller domain, and so that the newly found structure-derived motif would exist in every repeat. As the result, among 47 available genomes of unicellular eukaryotes we could not find a single instance of seven repeats with the motif. Several sequences contained three repeats, a predicted transmembrane segment, and a short cytoplasmic motif associated with some integrins, but otherwise differ from the metazoan integrin α subunits. Among the available bacterial sequences, we found five examples containing seven sequential metazoan integrin-specific motifs within the seven repeats. The motifs differ in having one Ca^2+^-binding site per repeat, whereas metazoan integrins have three or four sites. The bacterial sequences are more conserved in terms of motif conservation and loop length, suggesting that the structure is more regular and compact than those example structures from human integrins. Although the bacterial examples are not full-length integrins, the full-length metazoan-type 7-bladed β-propeller domains are present, and sometimes two tandem copies are found.

## Introduction

Integrins are large, heterodimeric cell-surface receptors that detect and transmit changes in mechanical forces resulting from interactions between a cell and the extracellular matrix [1]. Cell-cell and cell-matrix adhesion, mediated by integrins, play “key” roles in inflammation, cell development, and cell proliferation and differentiation [2], [3].

Vertebrates express at least 18 different integrin α subunits and eight β subunits, forming 24 α/β heterodimeric receptors in human [4]. Half of the integrin α subunits in human, namely α1, α2, α10, α11, αL, αM, αX, αD, and αE, contain an additional domain – a von Willebrand factor type-A (vWA) domain, inserted between the second and the third blades of the N-terminal 7-bladed β-propeller domain, referred to as either the αA domain [5] or the αI domain [6]; the β-propeller domain is found in all integrin α subunits. All integrins with an inserted αI domain bind their natural ligands via the metal ion dependent adhesion site (MIDAS) of the αI domain and appear to have arisen within integrin α subunits around the divergence of the first chordates since they are found in some integrin α subunits from tunicates but not from the earliest-diverging deuterostomes, e.g. the echinoderms [7]–[9].

The remaining nine α subunits, α3, α4, α5, α6, α7, α8, α9, αV, and αIIb, do *not* contain the αI domain. The Arg-Gly-Asp (RGD) sequence, present in the integrin ligands fibronectin and laminin, is one of the very first motifs that was found to be recognized by integrins [10], and it was shown that all known RGD-recognizing integrins lack the αI domain. In these integrins, the carboxylate side-chain of the aspartate residue of the RGD peptide binds to the Mg^2+^ coordinated by MIDAS of the I-like βA domain present in the β subunit, while the arginine side chain directly binds to the N-terminal β-propeller domain of the α subunit [11]. Thus, the N-terminal β-propeller domain either directly participates in ligand recognition, as with all integrins having an α subunit lacking the αI domain, or it incorporates a ligand-binding αI domain budding from the β-propeller domain through which it recognizes the ligand [4], [12], [13]. In either case, the β-propeller domain of the α subunit forms key stabilizing interactions with the βA domain of the β subunit.

A study of 90 integrin α subunits and 57 β subunits from 26 different metazoan species, ranging from *C. elegans* to *H. sapiens* and including sequences from the tunicate *C. intestinalis* and the pufferfish *T. rubripes*, has shown that orthologues of the human α subunits and β subunits of the integrins are highly conserved in bony vertebrates [7], whereas earlier-diverging integrins from sponges, nematode, insects through to the earliest chordate integrins are not human orthologues [7], [9]. However, both the α and β subunits of integrins have been detected throughout the metazoans, including sequences from some very early diverging species such as the sponge *Geodia cydonium*.

A cell adhesion system involving proteins with the RGD sequence may have already existed in protozoa since a GRGDSPK peptide but not the GRGESPK peptide caused substratum-detachment of cells of the marine amoeba *Neoparamoeba aestuarina* [14]. Recently, Sebé-Pedrós et al. have reported that genomes of two unicellular eukaryotic organisms, *Capsaspora owczarzaki* (a unicellular organism diverging just prior to metazoans) [15] and in *Thecamonas trahens* (formerly *Amastigomonas sp.*) contain domain sequences that appear to have the hallmarks of α and β subunits of integrins [16].

The first report of similarities between bacterial sequences and integrin sequences was made over ten years ago, when May and Ponting reported that an automated run of PSI-BLAST [17] showed sequence similarity between the cytoplasmic portion of the human integrin β4 subunit (residues 735–1125; the human β4 subunit has a large cytoplasmic domain in comparison to other integrin β subunits) and a region encoding a hypothetical protein from the cyanobacteria *Synechocystis* sp. PCC6803 [18]. The authors also reported sequence similarity between another sequence from the same bacterium and several β-propeller repeats from different integrin α subunits. Shortly after, Jenkins et al. showed homology between *Planctomycetale* bacteria *Gemmata obscuriglobus* and integrin αV, including the putative Ca^2+^-binding region common to integrins [19].

In the course of our own search of the available sequence data for sequences matching human integrin α and β subunits, we identified several matching sequences in bacteria that aligned surprisingly well with portions of the integrin subunits [9]. For example, a sequence from the cyanobacterium *Trichodesmium erythraeum* is homologous with more than 450 residues of integrin β-subunit which includes the amino-terminal βA-domain but other domains such as the EGF repeats and transmembrane segment were not present in the sequence [9]. Sequences also matched with the integrin α subunits. On closer inspection of the alignments it is was clear that the bacterial sequences matched the repeating units corresponding to blades of the β-propeller domain, but that the reported sequences did not include the trans-membrane domain, and the Thigh, Calf-1, or Calf-2 domains were not recognizable in the sequences. However, it remained unclear whether the repeating units that were observed in bacteria would indicate the presence of an integrin-like 7-bladed β-propeller or a β-propeller formed from a different number of blades, or if they represented an entirely different fold.

As defined in the SCOP database, the N-terminal β-propeller domain of integrin α subunits has the 7-bladed β-propeller fold [20]. The integrin β-propeller domain is only one of the fourteen superfamilies of proteins with the 7-bladed β-propeller fold. In addition to the 7-bladed β-propeller fold, 4-, 5-, 6- and 8-bladed β-propeller folds also exist and each fold type contains one or more protein superfamilies (see SCOP) [20]. The 7-bladed β-propeller fold, however, is represented by the largest number of solved structures deposited with the Protein Data Bank (PDB), with more than 115 reported protein structures [21].

Structures of three different integrins containing the β-propeller domain, all from human, have now been solved: αVβ3 [11], [22]–[24], αIIbβ3 [25]–[27], and αXβ2 [28]. Here, we have made an in-depth analysis based on the two highest-resolution X-ray structures: the ectodomains of human αVβ3 (3.10 Å resolution; PDB ID: 1JV2) [22] and αIIbβ3 (2.40 Å resolution; PDB ID: 2VDR) [25], as well as the sequences from the remaining 16 human α subunits. We identified the structural features that distinguish the human integrin-type 7-bladed β-propeller superfamily from the other 13 superfamilies having the 7-bladed β-propeller fold and demonstrate that the structural features of the human integrin-type 7-bladed β-propeller superfamily uniquely specifies a pattern of sequence conservation that can be used to identify sequences fulfilling the requirements for forming the structures.

Previously, it was already shown, that at least four non-integrin type of 7-bladed β-propeller domains as well as several 8-, 6- and 4-bladed β-propeller domains were found in bacteria [29], [30]. Herein we present strong evidence that bacterial sequences encode structures that are equivalent to the 7-bladed β-propeller found in human integrins.

## Results and Discussion

### Consensus sequence repeats define the seven blades of the integrin alpha N-terminal domain

There are two reported descriptions of a repeating consensus sequence for human integrin β-propeller domains, but both define the same basic consensus sequence: (1) the “FG-GAP repeat” sequence motif [31], [32], and (2) the “Cage” sequence motif [22].

The FG-GAP repeat motif was defined based on sequence similarities, and a structural model of the integrin β-propeller domain proposed a repeating structure, where each “blade” contains a 4-stranded β-sheet with a Phe-Gly (FG) pair in the first strand *plus* a Gly-Ala-Pro (GAP) tripeptide in the second strand [31], [32]. The Cage motif was defined by the φφGφX_13–20_PX_2–15_GX_5–8_ (φ, aromatic residue; G, glycine; X, any residue, P, proline) consensus sequence reported with the X-ray structure of the human αVβ3 ectodomain [22]. The first glycine residue of the Cage motif and glycine of the FG pair of the FG-GAP repeat motif are the same residue, as are the proline residues. No 3D structural motif has, however, been defined.

When, in an earlier sequence-based search [9], bacterial sequences were identified with what appeared to be the repeated consensus sequence patterns, it was unclear whether they were truly of the integrin type, similar to one of the other 13 β-propeller fold superfamilies, or perhaps representing a novel superfamily having the same fold. Since 3D structure is well-known to be more conserved than sequence, and the information derived from analysis of known 3D structures places additional constraints on a consensus sequence motif, we made a detailed comparison of the two independent higher-resolution β-propeller domain structures from human integrins.

### Defining a structure-based motif for blades of the human-type β-propeller domain

In addition to sequence data on all of the human integrin α and β subunits, we have considered the high-resolution X-ray structures for the human integrin ectodomains: αVβ3 [22] and αIIbβ3 [25]. Both of the αV and αIIb subunits contain an N-terminal domain with the 7-bladed β-propeller fold, with a 4-stranded β-sheet having the FG-GAP/Cage sequence motifs repeated in each of the 7 blades. Unlike the lower resolution αXβ2 structure [28], neither integrin has an inserted I domain in the β-propeller domain. The “integrin alpha N-terminal domain” is only one superfamily out of 14 having the all-β class 7-bladed β-propeller fold (see SCOP) [20].

In this study, we considered only the sequences and structures of the β-propeller domain and excluded other regions including the I domain sequence that buds out of the β-propeller in 9 of the 18 human integrin α subunits. In order to more fully-characterize the repeating unit, we have made detailed comparisons of the sequences and structures in order to define a structural motif.

Any structural motif has two major features: (1) it includes segments of the polypeptide chain with identical secondary structure, which (2) interact in an identical way to fulfill their function. Thus, based on the definition of the motif we should be able to identify key essential features that define each blade of the integrin domain, differences with the other reported family members, and see if the sequence patterns in the bacterial sequences or other non-integrin sequences support the same structural requirements as the integrin β-propeller domains.

**Figure 1:**
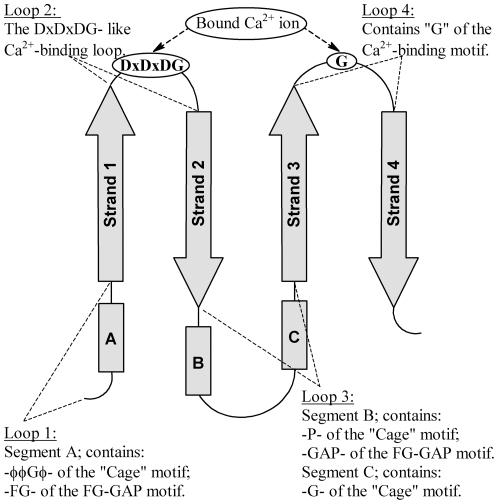
Schematic of the four-stranded super-secondary structure of a single blade, which is repeated seven times to form the β-propeller domain of the integrin α subunit. Segments A, B and C from the loops adjacent to β-strands 1, 2 and 3 show the location of key amino acids of the two defining sequence motifs: φφGφX_13–20_PX_2–15_GX_5–8_ of the Cage motif, and the FG-GAP (pfam01839) motif. The position of the calcium-binding motif, which is found in blades 5 through 7 of all known integrins, and in blade 4 of integrins αIIb, αV, α5 and α8, is also shown in the loop regions between β-strands 1 and 2 and β-strands 3 and 4 (see Table S1 for reference).

**Figure 2:**
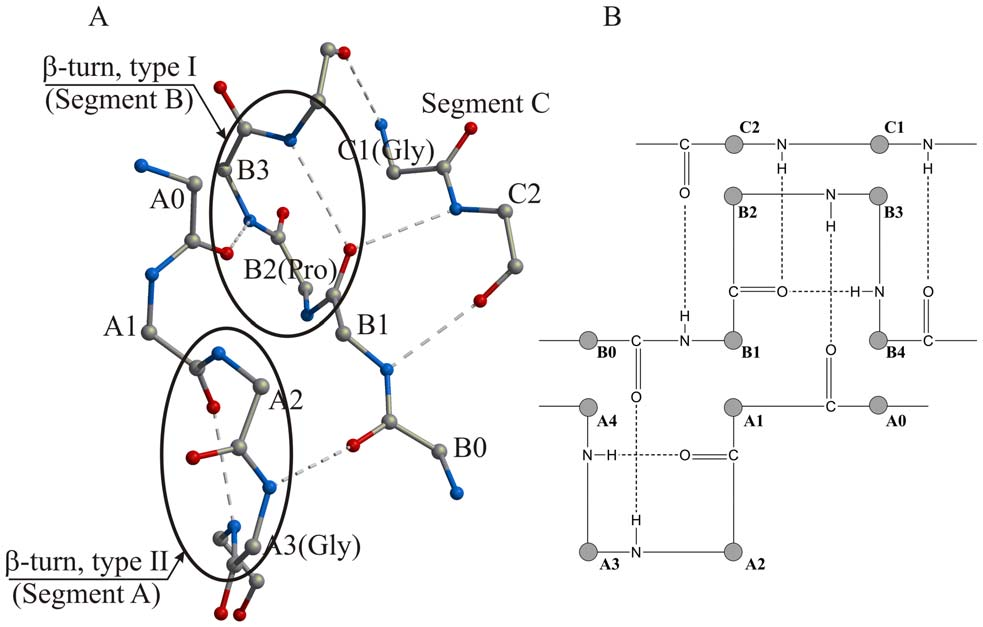
Three-dimensional (A) and schematic (B) representations of segments A, B and C (Table S1), which incorporate key amino acids from the Cage and the FG-GAP consensus repeat motifs. The three segments interact by means of a network of specific hydrogen bonds, which are identical for all blades from the β-propeller structures of αVβ3 and αIIbβ3. In addition, the three segments have a conservative secondary structure, where segments A and B contain β-turns of type II and I, respectively, thus requiring residue A3 (Gly170 in αIIb) to be nearly always glycine and residue B2 (Pro186 in αIIb) to be a proline. The network of conserved hydrogen bonds among segments A, B and C together with the calcium-binding motif join the loops from both sides of a four-stranded blade, resulting in a very compact blade structure. Numeration of amino acids A0–A4, B0–B4 and C1–C2 corresponds to those shown in Table S1. The structure in (A) is based on the structure of the αIIb subunit (PDB ID: 2VDR).

Each blade from the integrin-type β-propeller domains consists of four antiparallel β-strands and four loop regions; in Figure 1, segment A is part of loop 1 and segments B and C are part of loop 3. In every blade of the 7-bladed β-propeller, the key residues of the Cage motif reside within segments A, B and C, whereas those defined by the FG-GAP motif reside within segments A and B (Figure 1). Within a single blade, segments A, B and C interact with each other by means of a network of hydrogen bonds (Figure 2), which link five amino acids from A (residues A0–A4), five from B (residues B0–B4), and two from C (residues C1, C2).

**Figure 3:**
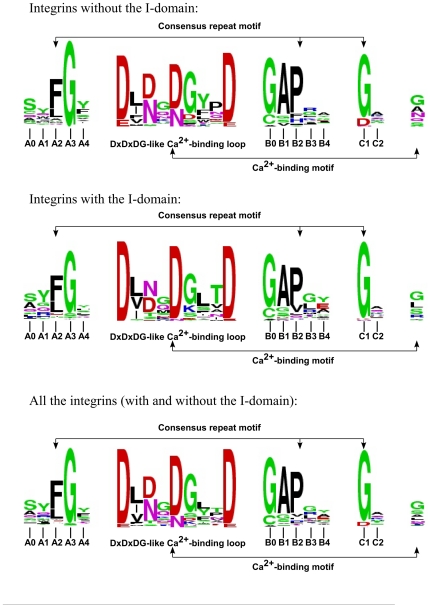
A WebLogo analysis of amino acid side chains of the consensus repeat motif incorporated into segments A, B and C and the calcium-binding motif from each of the seven blades of all of the human integrin sequences. Integrins with and without the I domains were analyzed together and separately.

These positions within the three segments, tabulated for all 7 blades from the β-propeller domains of the 18 human integrin α subunits (Table S1), are highly conserved (Figure 3). In addition to a high level of sequence conservation across the blades, the blades from the structures of the β-propeller domains from the human αV and αIIb subunits show that the number, geometry and orientation of the hydrogen bonds that join segments A, B and C, are identical in each of the seven blades from the two structures (Figure 2). The secondary structure of the segments A, B and C is also similar in all seven blades of the β-propeller domains of integrins αV and αIIb; segment A contains a classical type II β-turn, and segment B contains a classical type I β-turn.

**Figure 4:**
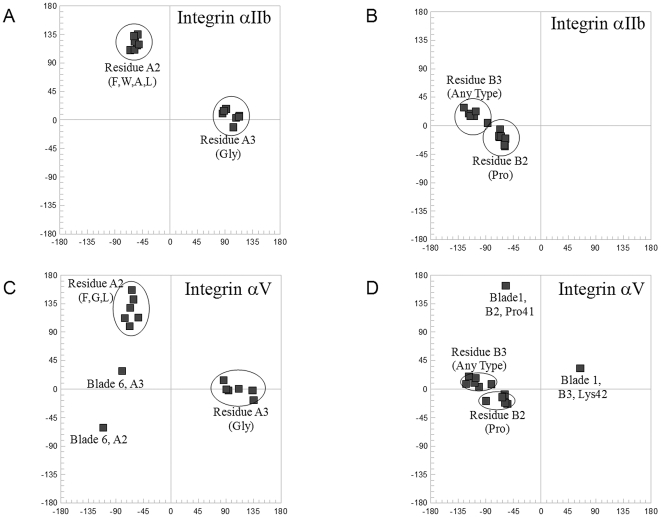
Fourteen pairs of torsion angles of amino acids A2 and A3 (segment A), and B2 and B3 (segment B), from the αV and αIIb subunits of two integrin structures are summarized on four Ramachandran plots (A) through (D). Values of the torsion angles ϕ and ψ and the amino acid composition for the second and third amino acids from the conserved β-turns within the segments A and B correspond to those of classical β-turns of type II (amino acids A2 and A3) and type I (amino acids B2 and B3). There are two exceptions: (C) in the 6th blade of integrin subunit αV segment A contains a non-standard β-turn instead of a type II β-turn; and (D) in the 1st blade of the αV subunit segment B contains a type II β-turn instead of a type I β-turn. Residue A3 is a glycine and residue B2 is a proline in almost all β-turns, which is in accordance with the β-turn type.

A type II β-turn is a four-residue turn characterized by the following three criteria (for properties of β-turns see Richardson [33], and Hutchinson and Thornton [34]): (1) torsion angles ϕ2 = −60°, ψ2 = +120°, ϕ3 = +90°, ψ3 = 0°; (2) the residue at position 3 is almost always a glycine; and (3) a C-O···H-N hydrogen bond is formed between the main-chain oxygen atom of the residue at position one and the main-chain nitrogen atom of the residue at position four. The conservation of the type II β-turn in the β-propeller blades of integrins is shown via a Ramachandran plot for residues A2 and A3 (i.e. the second and third residues of the turn; Figure 4A), demonstrating that residues A2 and A3 from all of the blades in both structures except blade 6 from the integrin αV subunit (Figure 4A) have torsion angles corresponding to a classical type II β-turn. Residues A2 and A3 from blade 6 of the integrin αV subunit are among the residues noted by the authors to deviate from expected values; the structure was solved at a fairly low resolution (3.10 Å, PDB ID: 1JV2) (Figure 4C). The other two criteria of the type II β-turn, namely presence of a glycine and a stabilizing hydrogen bond, are also fulfilled with no exceptions.

As seen in the structures of the human αV and αIIb subunits, segment B corresponds to a classical type I β-turn with the consensus sequence APXX (A, alanine; P, proline; X, variable), with torsion angles near ϕ_B2_ = −60°, ψ_B2_ = −30°, ϕ_B3_ = −90°, ψ_B3_ = 0° (Figure 4B). The conserved proline residue is found in most of the blades of the β-propeller domain in each of the 24 human integrin sequences (position B2 in Figure 2; Table S1). Of the 14 examples from the human αV and αIIb subunits, there is only one exception: blade 1 from the αV subunit has a non-standard conformation, ϕ_B2,Pro41_ = −57°, ψ_B2,Pro41_ = 163°, ϕ_B3,Lys42_ = 64°, ψ_B3,Lys42_ = 32°, which is more typical for a type II β-turn (Figure 4D).

As a consequence of the structural requirements of the type II and type I turns, two conserved residues common to the Cage and FG-GAP motifs, glycine and proline, are respectively found with high frequency at positions A3 and B2. It is due to the structural requirements that both residues are conserved in the sequence motif descriptions. In addition to the hydrogen bond stabilizing each turn type, there are 5 additional hydrogen bonds between main-chain amino and carbonyl groups that link segments A, B and C to form a rigid unit (Figure 2). These segments together with the β-sheet formed from strands 1–4 form a blade linked by a network of main-chain hydrogen bonds; the blade is further stabilized by binding Ca^2+^ at the opposite end of the blade to segments A–C.

Glycine in the type II turn and proline in the type I turn are conserved but not invariant (Figure 3). Our analysis shows that in human integrins, the probability for a glycine to occupy the third position in the first β-turn (position A3) is 89%, while the probability for a proline to occupy the third position in the second β-turn (position B2) is 71%. These probabilities show that these two types of amino acid are at least 100 times more frequent than any other type of amino acid taken separately. These data are in good agreement with the overall analysis of β-turns from unrelated protein structures [34], where among 405 type II β-turns and 1231 type I β-turns, the probability to find glycine at position A3 is 75% (13 times more frequent than any other amino acid) and the probability to find proline at position B2 is 17% (2 times more frequent). As a result, the consensus repeat sequence motif corresponds to a specific 3D H-bonded structural motif interacting by means of a network of hydrogen bonds (Figure 2).

### Ca^2+^-binding motifs stabilize the structure of several integrin blades

**Figure 5:**
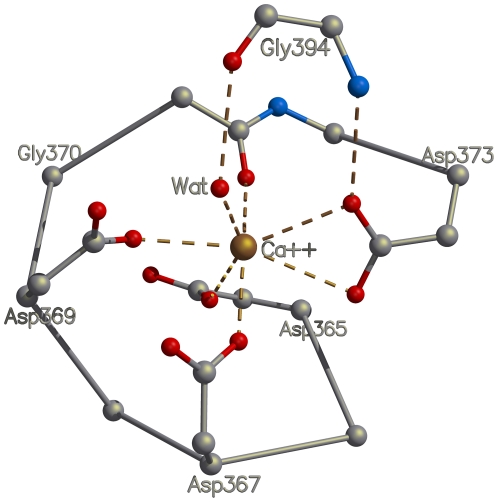
The calcium-binding motif present in several blades of the human integrin β-propeller domain is the well-known DxDxDG-like Ca^2+^-binding loop. In the integrin structures, the calcium-binding motif has an identical structure and similar mode of calcium binding. In every calcium-binding blade, conserved amino-acid side chains from the loop between β-strands 1 and 2 coordinate a calcium ion by means of a network of ionic interactions, while the main-chain oxygen and nitrogen atoms of the amino acid from the loop between β-strands 3 and 4 interacts with the calcium cation through a conserved water molecule and a conserved side chain of an aspartate residue.

In the structures of the human αV and αIIb subunits, located at the opposite end of the blade from the FG-GAP/Cage consensus repeat, there are two loops joining the four β-strands that are involved in binding Ca^2+^ (Figure 1; Table S1). The Ca^2+^-binding motif is not present in all seven blades of the β-propeller domain. However, in those blades where the motif is present it is conserved and consists of the well-known “DxDxDG-like Ca^2+^-binding loop” (D, aspartate; in the 1,2-loop, Figure 1), present in a large number of unrelated Ca^2+^-binding proteins [35], plus a glycine residue (in the 3,4-loop) that coordinates via main-chain atoms a water molecule bound to Ca^2+^. Thus, in the integrins the motif for calcium binding is structural and is more complex than a DxDxDG-like Ca^2+^-binding motif, and serves to bridge the two loops (Figure 5). The motif is conserved across human integrins for blades 5, 6 and 7, and additionally, is also present in integrins αV, αIIb, α8 and α5 in blade 4 (Table S1). In the X-ray structures of αV and αIIb, Ca^2+^ ions are bound to all four of the Ca^2+^-binding loops. Consequently, in the integrins, the consensus repeat motif and the Ca^2+^-binding motif form two anchors at opposite ends of the β-sheet of the blade that stabilize the structure of the integrin β-propeller blades from both sides.

### The structural motif distinguishes the integrin β-propeller from 13 other superfamilies

The β-propeller domain of the integrin α subunits corresponds to one of the fourteen superfamilies of 7-bladed β-propeller fold classified in the SCOP database [20]. An examination of all 32 representative structures from the other 13 superfamilies was conducted and four specific characteristics distinguish the integrin β-propeller from the other superfamilies demonstrating that the structural consensus motif for a blade exists only in the integrin superfamily and is not present in the other 7-bladed β-propeller fold superfamilies:

Characteristic 1, the presence of the Cage motif: The Cage motif [22] is not present in any of the representative structures from the other 13 superfamilies. This can be seen by using a pattern value description of the Cage motif to check for the sequence motif with the PATTINPROT web-based service (PATTINPROT, http://npsa-pbil.ibcp.fr/). Moreover the conservation of the key glycine and proline residues was not observed in the 13 superfamilies.

Characteristic 2, the presence of type II and type I turns: Only in integrin α subunits, and not in the other 7-bladed β-propeller fold proteins, we observe the presence of two β-turns, one type II and one type I turn, within each blade. In the 32 representative structures from the other 13 superfamilies, 12 structures do not contain even one pair of β-turns within two adjacent segments A and B as seen in the integrins (see Figures 1 and 2). The remaining 20 representative structures (Table 1) contain a pair of β-turns in at least one of the seven blades, with a maximum of four examples seen only in the nucleoporin domain superfamily. Thus, with the exception of the integrin α subunits, there are no other structures from any of the superfamilies of 7-bladed β-propeller domains that have β-turns in the adjacent segments A and B in all seven blades.

**Table 1:** Presence of β-turns in segments A and B in the seven blades of 20 representative structures of proteins that have the 7-bladed β-propeller fold (*excluding* the integrins).

	Blade/Turn type						
Protein Family	Blade 1	Blade 2	Blade 3	Blade 4	Blade 5	Blade 6	Blade 7
1. YVTN repeat:							
1L0Q_A: Segment A	−	−	−	−	−	−	Type I
1L0Q_A: Segment B	+	+	+	+	+	+	Type I
2. Quinohemoprotein amine dehydrogenase B chain:							
1PBY_B: Segment A	Type II'	−	+	−	−	−	−
1PBY_B: Segment B	Type VIa1	+	−	−	−	−	−
3. Nitrous oxide reductase, N-terminal domain:							
1FWX: Segment A	Type II	−	−	−	−	−	−
1FWX: Segment B	Type I	+	−	+	+	+	+
4. WD40-repeat:							
1NR0_A(domain I): Segment A	−	−	−	−	−	Type I	−
1NR0_A(domain I): Segment B	+	−	+	+	+	Type I	+
1NR0_A(domain II): Segment A	−	−	−	−	−	−	Type II
1NR0_A(domain II): Segment B	+	+	+	+	+	+	Type I
5. RCC1/BLIP-II:							
1A12_A: Segment A	−	−	−	−	Type I	−	+
1A12_A: Segment B	+	+	+	+	N/S	+	−
6. Clathrin heavy-chain terminal domain:							
1UTC_A: Segment A							
1UTC_A: Segment B	Type I	−	−	Type II	−	Type I	−
	Type II'	+	−	Type I	+	Type VIb	+
7. Peptidase/esterase ‘gauge’ domain:							
1H2W_A: Segment A	+	Type I	−	+	−	−	−
1H2W_A: Segment B	−	Type I'	−	−	+	+	−
8. Tricorn protease domain 2:							
1K32_A: Segment A	Type I	−	−	−	−	−	−
1K32_A: Segment B	Type I'	+	+	+	−	−	+
9. 3-carboxy-cis, cis-mucoante lactonizing enzyme:							
1JOF_A: Segment A							
1JOF_A: Segment B	Type I	+	N/S	Type I	−	+	+
	Type II'	−	Type I	Type I	+	−	−
10. Putative isomerase YbhE:							
1RI6_A: Segment A	−	−	Type II	N/S	−	−	−
1RI6_A: Segment B	−	+	Type I	N/S	−	+	+
11. Sema domain:							
1OLZ_A: Segment A	−	−	Type I	+	−	−	−
1OLZ_A: Segment B	−	+	Type I	−	−	+	+
12. Oligoxyloglucan reducing end-specific cellobiohydrolase:							
1SQJ_A: Segment A							
1SQJ_A: Segment B	−	N/S	+	−	Type II	−	−
	−	Type I	−	−	Type VIb	−	−
13. Nucleoporin domain:							
1XKS_A: Segment A	Type I	N/S	−	−	Type I	+	Type I'
1XKS_A: Segment B	Type II'	Type I	+	−	Type I	−	Type I

“+”, a β-turn is present; “−”, a β-turn is absent. The β-turn type is indicated when simultaneously present in both segments A and B, similar to what is seen in integrins. N/S, the type of the β-turn is not specified.

Characteristic 3, a hydrogen bonding network linking segments A, B and C: Only in the integrins, in each of the seven blades, the two β-turns residing in the segments A and B are closely aligned and interact with each other via a network of hydrogen bonds, as shown in Figure 2. Although two β-turns are found on adjacent segments A and B in some blades in the other representative structures (Table 1), in the X-ray structures these turns are without exception located beyond H-bonding distance.

Characteristic 4, the presence of a Ca^2+^-binding motif: The Ca^2+^-binding motif formed from two adjacent loops only found in the integrin-type β-propeller domain.

Characteristic 5 (non-specific), a non-sequential 4th strand in blade 7 arising from the sequence N-terminal to blade 1 of the domain. This is seen in the structures of the β-propeller domain from the human αV and αIIb subunits but it is not a feature unique to the β-propeller domain from the human α subunits.

The presence of four characteristics specific for the integrin-type β-propellers and one characteristic specific to the β-propeller fold in general, appears to uniquely define the blades of the integrin-type β-propeller domain. Furthermore, these features demonstrate that the repeating unit is an extensively stabilized structural motif, defined by sequence and structural features, which include the FG-GAP/Cage sequence motif forming two interlocked β-turns, the Ca^2+^-binding motif, as well as the four antiparallel strands and the extensive hydrogen bonding network.

### Search for the consensus repeat motif in bacteria

**Figure 6:**
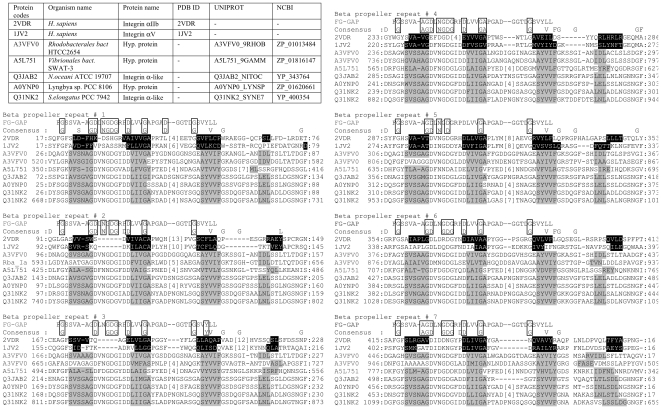
Sequence alignment of the β-propeller domains from the human integrin αV and αIIb subunits and seven sequences from five different bacteria, where seven FG-GAP consensus repeat motifs (pfam01839) were identified. The secondary structure (β-strands of the seven blades) from the structures of αV and αIIb is shown with dark shading, while the secondary structure of the bacterial sequences, predicted by three different methods, PHD, PSIPRED and PROF (see Materials and Methods) is shown with light shading. The upper sequence shown in the alignment is the consensus Hidden Markov Model (HMM) signature sequence of the FG-GAP (pfam01839) motif, followed by the consensus sequence (designated Consensus) derived from the bacterial sequences shown in the alignment. Identical residues in the two consensus sequences are boxed.

For the purposes of this study, we established extremely restrictive criteria in order to identify bacterial sequences that are most similar to the human integrin β-propeller domain. First, we extracted all sequences matching at least one copy of the signatures for the human integrin-type β-propeller domain using Pfam (consensus sequence pfam01839) [36]. The pfam01839 signature motif defines a blade of a human integrin β-propeller domain and incorporates information on the secondary structure, as well as the signatures for the Ca^2+^-binding site and the consensus repeat that includes the FG-GAP/Cage sequence. As the result, 1093 sequences were identified, of which 473 are eukaryotic sequences and 620 are bacterial sequences. Among the 1093 sequences, 9 eukaryotic and 58 bacterial ones were redundant or annotated as “obsolete” by the UniProtKB protein database (http://www.uniprot.org). The remaining 464 eukaryotic sequences and 562 bacterial sequences are given in the supplementary material section as two separate fasta files, eukaryote_464_init_record.fas and bacterial_562_init_record.fas, for eukaryotic and bacterial sequences respectively. The taxonomic groups and the numbers of identified sequences are shown in Table 2. Each of the 562 bacterial sequences was inspected for the presence of at least seven repeats of the FG-GAP/Cage consensus signature, both significant and insignificant (as defined in Pfam), using the Pfam “sequence search” procedure. As the result, we identified 229 such sequences, which we give as the supplementary fasta file (bacterial_229_with_7_signatures.txt). Each of the 229 sequences in the file contains additional information about the number of identified signatures. Some sequences had 14 total signatures, suggesting the presence of tandem copies of the putative domain. Presence of a Pfam consensus signature does not automatically mean the presence of the FG-GAP/Cage motif. From the 229 identified sequences we removed all the sequences, where the Pfam consensus signature did identify protein sub-sequences, which were shorter than that is required to incorporate all structural elements of the structure-based motif described earlier. As the result, we obtained 35 sequences from 21 different bacteria (given in the supplementary material section as a file bacterial_35_with_7_full_length_motifs.txt), which contained seven full-length segments able to incorporate the structure-based motif for blades of the human integrin-type β-propeller domain described earlier, and where each of the seven full-length segments contained the Pfam-defined FG-GAP/Cage consensus signature. The 35 sequences were then scrutinized in order to identify and confirm the presence of the consensus FG-GAP/Cage motif in each of the seven repeat signatures. This led to 9 sequences from five different bacterial species, of which 5 have been aligned with the structural alignment of the repeats found in human integrin subunits αV and αIIb (Figure 6). The alignment contains one representative (A3VFV0_9RHOB) from *Rhodobacteriales* HTCC 2654 where there are 4 sequence entries, each with 14 repeats. *Nitrococcus oceani* ATCC 19707 has two sequence entries with seven repeats; one representative sequence, Q3JAB2_NITOC, is present in the alignment of Figure 6. The alignment of the repeats from all 9 sequences, including the two sets of seven repeats found in the four *Rhodobacteriales* HTCC 2654 entries and the sequence from *Synechococcus elongatus* PCC 7942 (Q31NK2_SYNP7), is presented in the Supplementary Material.

**Table 2:** Taxonomic groups and the identified number of sequences deposited to the UniProtKB database that contain domain architectures similar to (and including) the integrin α (β-propeller) superfamily.

Eukaryotes (464):			
	Chordates (323):		
		Vertebrates (308):	
			Mammals (232);Bony fishes (51);Amphibians (18);Birds (7);
		Lancelets (12);Tunicates (3).	
	Arthropods (77):		
		Insects (74);Ixodida (3).	
	Echinoderms (6);Cnidarians (8);Nematodes (6);Placozoans (2);Sponges (1);Other Eukaryotes (41).		
Bacteria (562):			
	Proteobacteria (123):		
		G-proteobacteria (33);D-proteobacteria (50);B-proteobacteria (6);A-proteobacteria (31);Other proteobacteria (3).	
	Actinobacteria (149);Cyanobacteria (96);Bacteroidetes (58);Planctomycetes (20);Acidobacteria (33);Spirochetes (9);Firmicutes (12);Chloroflexi (10);Lentisphaerales (5);Chlorobi (5);Verrucomicrobia (39);Other Bacteria (3).		

For the 5 sequence entries (seven sets of seven repeats), the individual secondary structure predictions and the consensus predicted secondary structure made using three different methods, PHD [37], PSIPRED [38] and PROF [39] (see Materials and Methods), coincided well with that of the known human β-propeller domain structures, with an identical distribution of β-strands as seen in the structures of integrins αV and αIIb (Figure 6). In the X-ray structures, the fourth β-strand in the final seventh blade of the β-propeller domain arises from sequence located just before the sequence of the first blade. In this way, the N- and C-terminal ends of the β-propeller domain in the integrins are locked together. In each of the bacterial sequences a short β-strand was predicted adjacent to the N-terminus of the first β-strand of the first blade of every 7-blade repeat. The N-terminal sequences observed and predicted to be contributing to the fourth strand of the seventh blade are shown at the very end of the alignment in Figure 6.

Conserved residues in the bacterial sequences, labeled “Bact_Consensus” and computed for each individual blade, are shown in Figure 6 in comparison with the consensus Hidden Markov Model signature sequence of the “FG-GAP” (pfam01839) motif computed over all blades of the β-propeller domain from integrin α subunits. Both consensus sequences show that the three key glycine residues from the FG-GAP/Cage motif are highly conserved. The key proline present in many of the integrin repeats is also present in repeats from the bacteria. More surprisingly, the comparison of the two consensus sequences showed that the Ca^2+^-binding site exists and it is conserved in each of the bacterial sequences but, in contrast to the human integrin β-propeller domains, the signature is present in all 7 blades. This strongly suggests that the bacterial sequences form β-propeller domains, quite similar to the β-propeller domains of the human integrin α subunits, and that the Ca^2+^-binding motif is exploited even more frequently than in the integrins to form one of the two anchors that stabilizes each blade of the β-propeller. This in itself suggests that the bacterial sequences might form a more regular, disk-like three-dimensional than those of the human integrins. It also would be consistent with the idea of seven tandem repeats arising first in bacteria and then later being adapted for use in the integrins with a loss of some Ca^2+^ binding sites.

The analysis reported here demonstrates that the bacterial sequences do not share the features of the *other* 13 families of β-propeller fold proteins, but clearly display the sequence and implied structural features of the 7-bladed β-propeller domains of the integrins. The similarities are striking and suggest a common origin of sequences derived from an approximately 60-residue repeat unit. In comparison to human integrins, these bacterial sequences have retained more common features within each repeat, i.e. a complete set of seven Ca^2+^-binding sequences and more consistent lengths of the loop regions, which is consistent with the notion of the bacterial sequences still retaining features of an ancestral form of the β-propeller domain that was later exploited in the integrin α subunits apparently arising early in or prior to the origins of multicellular organisms.

One can not help but ask what possible functions the integrin-like β-propeller domains have in bacteria. In the integrins the β-propeller domains interact with the βA-like domain of the integrin β-subunit. Ligands bind either to this complex (see, e.g. the structure of αVβ3 ectodomain with RGD peptide) [11] or to an αI domain budding out from the 7-bladed β-propeller domains (see for example, the structure of the α2I domain with bound collagen-like peptide) [40]. The integrins are also membrane-bound proteins, and the bacterial sequences that we have retrieved here do not have hydrophobic sequences consistent with a transmembrane helix that could anchor the protein to a membrane. In addition to being more uniform across the 7 repeats than the integrin sequences, some bacterial sequences have two closely spaced sets of 7 repeats, suggesting they would correspond to tandem 7-bladed β-propeller domains.

For the bacterial proteins any functional role would be limited to the cytoplasm. In integrin β-propeller domains Ca^2+^ may play a stabilizing structural role and Ca^2+^ levels are high outside of cells, thus the sites should always be occupied. Within cells, Ca^2+^ is generally at very low concentrations, thus giving it a unique role as a secondary messenger in signaling. In bacteria, calcium plays an increasingly recognized role in signaling [41], [42] and other critical cellular processes [43]–[45]. The bacterial domains have 7 repeated highly conserved Ca^2+^-binding motifs, sometimes duplicated in the same sequence, and it likely these sites are functional, but what role they might play in bacteria is unclear at this time. The high similarity between the bacterial domains and metazoan integrins and presence of the key features required for the structure, strongly suggest that the presence of this domain in modern bacteria belies the origin of β-propellers in the integrins. A key question concerns the basis for the apparently different functions in bacteria to those arising in the integrins in multicellular organisms.

### Integrins, β-propellers and origins of multicellularity

Integrins are present in species that represent the earliest metazoans. We have made a comprehensive search for integrin-type β-propeller domains from sequences of unicellular eukaryotes, including those submitted to sequence and protein databases as well as those that are located with current genome-sequencing projects. There are 47 on-going genome projects of eukaryotic unicellular organisms with identified genomic sequences at different stages, from which there are 38 projects where protein sequences have been deposited (NCBI Genome: http://www.ncbi.nlm.nih.gov/sites/genome), and nine others that include *C. owczarzaki* and *T. trahens* (Sebé-Pedrós et al. reported matches in these two species) [16].

The integrin α subunit-like sequence from *T. trahens* (formerly *Amastigomonas sp.*; sequence AMSG_06621.1) has three FG-GAP repeatsand the sequence record we obtained from the Broad Institute “Origin of Multicellularity” project did not have the transmembrane sequence. However, the sequences from *Capsaspora* have three repeats, a predicted transmembrane segment, and an adjacent KXGFFXR integrin motif. In contrast, sequence matches between true integrin β-propeller domains and domains observed in bacteria match all seven blades and the alignment is excellent throughout, albeit the latter examples additionally contain Ca^2+^-binding motifs in all seven blades. Aside from the sequences reported by Sebé-Pedrós et al. no clear sequentially-repeated FG-GAP motifs were detected within the 47 genomes [16].

*Ectocarpus siliculosus* is a multicellular brown algae and phylogenetic studies place *this species* among the heterokonts (stramenophiles), which are eukaryotes that also include a large number of unicellular diatoms, and whose origin of multicellularity occurred *independently* from the metazoans [46]. The recent sequencing of the genome of *Ectocarpus* has identified several “integrin-like” sequences [46]. One sequence of 348 residues (CBJ33612.1) matches well the N-terminal half of the 788-residue human β3 subunit (e.g. 23% sequence identity over residues 99–358; human β3 numbering), with excellent matching of the MIDAS segment. An α subunit-like sequence (CBN77719.1; 1105 residues) aligns with few gaps to the 1048-residue human integrin αV subunit (ITAV_HUMAN): percentage identity ranging from 41% over residues 245–500 (human αV numbering) to 17% sequence identity over the entire 1012-residue alignment; the 7 blades of the β-propeller domain align with each other and even the 4 Ca^2+^-binding motifs within *Ectocarpus* align to the 4 motifs found in the human αV subunit. Differences, however, appear towards the C-terminus: there is a glycine and alanine-rich low complexity region in the *Ectocarpus* protein, followed by an alanine and proline-rich region “matched” to the transmembrane segment of the αV subunit; the adjacent cytoplasmic KXGFFXR motif found in αV is not present in *Ectocarpus*, but this motif is also absent in some integrin α subunits, too.

Thus, in two independent lines of multicellular organisms, metazoans and heterokonts, the β-propeller is conserved to the point of having Ca^2+^-binding sites in the corresponding blades, and there are strong hints of both α-like and β-like integrin subunits in the heterokont *Ectocarpus*. The current scope of genomic sequence data is obviously sparse and incomplete. Nevertheless, these observations do raise some fundamental questions with regard to the origin and evolution of integrins and their role in the origin of multicellularity, suggesting that a *common* ancestor of both multicellular lines contained some if not all of the features of the integrin α and β subunits, including integrin-like β-propeller domains and βA domains. The observation of complete and highly-conserved integrin-type β-propeller domains in bacteria, described here, suggest that the origin of domains in integrins are indeed prokaryotic, giving rise to the components of the present-day integrin subunits. However the link between having individual domains in prokaryotes and their functions, and their integration into integrin subunits in multicellular organisms remains obscure. In metazoan integrins, the β-propeller domain of the α subunit is directly or indirectly (via the inserted A domain) involved in binding external ligands, but it is always involved in binding the βI-like domain of the β subunit. The α subunit protrudes from the exterior membrane surface where Ca^2+^ levels are typically much higher than in the cytoplasm; the highly-conserved calcium-binding sites of the β-propeller and their key structural role near the ligand and subunit-subunit binding sites explain why the formation of the integrin αβ heterodimer capable of binding a specific ligand is calcium-dependent. Although the function and cellular location of the β-propeller domains in bacteria is currently unknown, if these domains are confined to the cytoplasm, then perhaps they have a role related to calcium signaling or storage. Clearly, further genome sequencing combined with experimental studies aimed at establishing a functional role for these integrin-type domains in non-integrin sequences is needed in order to elucidate the linkage the establishment of the present-day integrin subunits from the putative origin in single-cell organisms.

## Materials and Methods

### Determination and Analysis of Protein Folds, Sequential and Structural motifs

The UniProt database (http://www.uniprot.org/) and the NCBI sequence database (http://www.ncbi.nlm.nih.gov/) were used as the initial sources of protein sequence and functional information. These two databases, particularly the UniProt database, are also widely used as sources of sequence data by structural databases and motif and pattern analysis computer programs, such as the Pfam (http://pfam.janelia.org/) [36]. All of the X-ray structures were taken from the PDB (http://www.rcsb.org) [21]. Protein folds, superfamilies and families were assigned according to the SCOP database (http://scop.mrc-lmb.cam.ac.uk/scop/) [20]. If a superfamily of 7-bladed β-propeller proteins contained several known structures, the structure with the best resolution has been used as a representative of the protein superfamily. Two different sequences, FG-GAP (pfam01839) and Cage [22], were used to define the consensus repeat sequence signature within the β-propeller domain of integrins. Secondary structure prediction of the retrieved bacterial sequences was based on the consensus alignment obtained using the results of three different computer-based secondary structure prediction methods: PHD from PredictProtein [37], PSIPRED [38] and PROF [39]. The φ and ψ angles of amino acids from the β-propeller repeats in the structures of the human integrins αV and αIIb subunits were calculated using SYBYL (Tripos Associates, Inc., St. Louis, MO). Structure visualization and structural analysis of geometrical parameters of interactions were done using SYBYL and BODIL [47]. The frequency of amino acids at positions within the β-propeller repeats were analyzed using the WebLogo server (http://weblogo.berkeley.edu/) [48]. Figures in this manuscript were produced with MOLSCRIPT V2.1 [49] and Raster3D V2.4b [50].

### Creation of the Bacterial Data Set

Using two signature sequences of the consensus repeat motif, pfam01839 and Cage, we have analyzed the UniProt and NCBI databases and retrieved 562 bacterial sequences that contain one or more instances of the consensus repeat signature sequence (the 562 bacterial sequences are given in the supplementary material section as a fasta file bacterial_562_init_record.fas). Next, among the 562 bacterial sequences, we have selected 229 sequences that contain seven or more consensus repeat signature sequences (the 229 sequences are given in the supplementary material section as a file bacterial_229_with_7_signatures.txt). Then, from the 229 identified sequences we identified 35 sequences from 21 different bacteria that contained seven full-length segments able to incorporate the structure-based motif for blades of the human integrin-type β-propeller domain (see Section: Search for the consensus repeat motif in bacteria). The 35 sequences were then scrutinized in order to identify and confirm the presence of the consensus FG-GAP/Cage motif in each of the seven repeat signatures (the 35 sequences are given in the supplementary material section as a file bacterial_35_with_7_full_length_motifs.txt). This led to nine sequences from five different bacterial species, which have seven consensus repeat motifs, similar to the β-propeller domains in human integrin α subunits (Figure S1). These five representative bacterial sequences were further analyzed with several secondary structure prediction methods. Predicted secondary structure and position of the consensus repeat motifs within the secondary structure elements were compared with those of known structures of human integrins and shown in Figure 6.

## Supporting Information

Figure S1 Alignment of nine sequences from five different bacterial species, which have seven full-length consensus repeat motifs, similar to those found in the β-propeller domains from the human integrin α subunits. For the nine sequences, the predicted secondary structure by three different methods, PHD, PSIPRED and PROF, coincided with the secondary structure of known human integrins.
(TIF)

Table S1 Localization of the FG-GAP/Cage motif and the Ca^2+^-binding motif within each of the seven blades of β-propeller domains of the human integrin α subunits.
(DOC)
