# Supplementary figures and images for: Conservation of the Human Integrin-Type Beta-Propeller Domain in Bacteria
Source: PLoS One. 2011 Oct 13;6(10):e25069. doi: 10.1371/journal.pone.0025069 (PMC3192720; doi:10.1371/journal.pone.0025069)

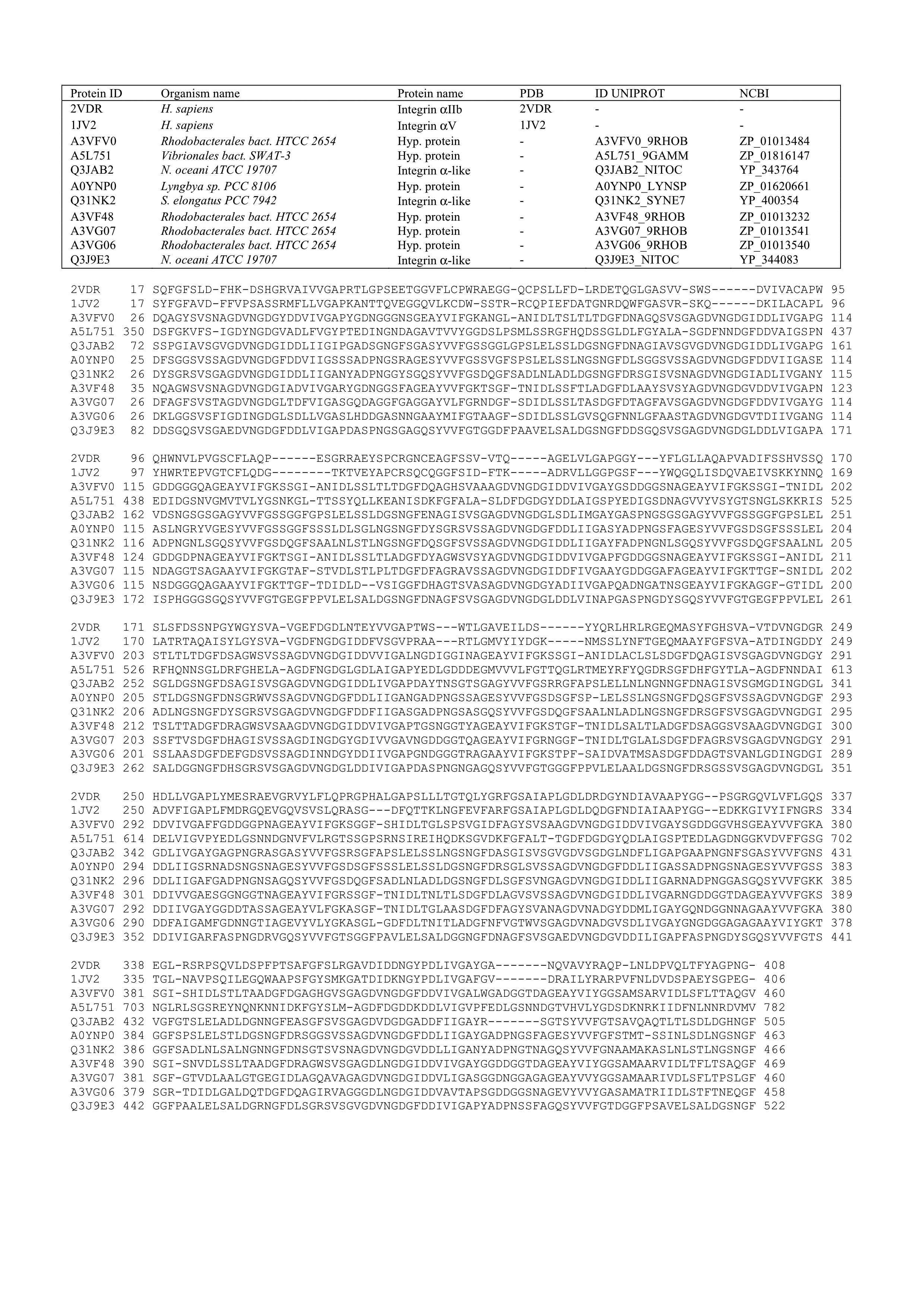

Supplement: Figure S1 — Alignment of nine sequences from five different bacterial species, which have seven full-length consensus repeat motifs, similar to those found in the β-propeller domains from the human integrin α subunits. For the nine sequences, the predicted secondary structure by three different methods, PHD, PSIPRED and PROF, coincided with the secondary structure of known human integrins. (TIF) [file pone.0025069.s001.tif]
