# Supplementary material for: Conservation of the Human Integrin-Type Beta-Propeller Domain in Bacteria
Source: PLoS One. 2011 Oct 13;6(10):e25069. doi: 10.1371/journal.pone.0025069 (PMC3192720; doi:10.1371/journal.pone.0025069)
Supplement: Table S1 — Localization of the FG-GAP/Cage motif and the Ca2+-binding motif within each of the seven blades of β-propeller domains of the human integrin α subunits. (DOC) [file pone.0025069.s002.doc]

Table S1. Localization of the FG-GAP / Cage motif and the Ca^2+^-binding motif within each of the seven blades of β-propeller domains of the human integrin α subunits.

|  | A0-A4 Ca^2+^ B0-B4 C1-C2 Ca^2+^  (Strand 1) (Strand 2) (Strand 3) |
| --- | --- |
| “Cage”  “FG-GAP” | -φφGφ-  -P- -G-  -FG- -GAP- |
| Blade 1  (without  the  I-domain) | IIb  ^17^SQFGF^21^ (16) ---  ^38^GAPRT^42^ (8)  ^51^GG^52^ ---  V  ^17^SYFGF^21^ (17) ---  ^39^GAPKA^43^ (10) ^54^GQ^55^ ---  5  ^58^SFFGF^62^ (16) ---  ^79^GAPKA^83^ (10) ^94^GA^95^ ---  8  ^55^SYFGY^59^ (16) ---  ^76^GAPKA^80^ (10) ^91^GA^92^ ---  4  ^50^TLFGY^54^ (15) ---  ^70^GAPTA^74^ (11) ^86^GA^87^ ---  3  ^50^SLFGY^54^ (18) ---  ^73^GAPRE^77^ (11) ^89^GA^90^ ---  7  ^50^SLFGF^54^ (18) ---  ^73^GAPQA^77^ (11) ^89^GG^90^ ---  6  ^42^SLFGF^46^ (18) ---  ^65^GAPRA^69^ (11) ^81^GG^82^ ---  9  ^11^SFFGY^15^ (15) ---  ^31^GAPKA^35^ (11) ^47^GA^48^ --- |
| Blade 1  (with the  I-domain) | 11  ^39^AFFGY^43^ (15) ---  ^59^GAPLE^63^ (7)  ^71^GD^72^ ---  D  ^33^GGFGQ^37^ (12) ---  ^50^GAPLE^54^ (7)  ^62^GR^63^ ---  1  ^45^DMFGY^49^ (15) ---  ^65^GSPLV^69^ (7)  ^77^GD^78^ ---  E  ^11^ASLAL^15^ (17) ---  ^33^GAPFV^37^ (7)  ^45^DP^46^ ---  2  ^46^EQFGY^50^ (15) ---  ^66^GSPWS^70^ (7)  ^78^GD^79^ ---  M  ^32^RGFGQ^36^ (12) ---  ^49^GAPQE^53^ (7)  ^61^GS^62^ ---  X  ^35^AGFGD^39^ (12) ---  ^52^GAPQK^56^ (7)  ^64^GG^65^ ---  L  ^43^RHFGY^47^ (11) ---  ^59^GAPGE^63^ (4)  ^68^GS^69^ ---  10  ^39^AEFGY^43^ (15) ---  ^59^GAPWD^63^ (7)  ^71^GD^72^ --- |
| Blade 2  (without  the  I-domain) | IIb  ^91^QGLGA^95^ (11) --- ^107^CAPWQ^111^(16)^128^GS^129^ ---  V  ^92^QWFGA^96^ (11) --- ^108^CAPLY^112^(13)^126^GT^127^ ---  5 ^140^QWFGA^144^(11) --- ^156^CAPLY^160^(13)^174^GT^175^ ---  8 ^134^QWFGA^138^(11) --- ^150^CAPLY^154^(14)^169^GT^170^ ---  4 ^125^QWLGV^129^(14) --- ^144^CGHRW^148^(14)^163^GG^164^ ---  3 ^122^MWLGV^126^(13) --- ^140^CAHRY^144^(15)^160^GK^161^ ---  7 ^122^QWLGV^126^(13) --- ^140^CAHRY^144^(16)^161^GR^162^ ---  6 ^113^QWMGV^117^(13) --- ^131^CAHRY^135^(16)^152^GR^153^ ---  9  ^88^EWMGV^92.^(14) --- ^107^CAHRW^111^(13)^125^GF^126^ --- |
| Blade 2  (with the  I-domain) | 11 ^105^LGLSL^109^(11) --- ^121^CSPLW^125^(11)^137^GM^138^ ---  D  ^90^LGLTL^94^ (11) --- ^106^CGPTL^110^(11)^122^GS^123^ ---  1 ^113^MTFGS^117^(12) --- ^130^CGPLY^134^(11)^146^GI^147^ ---  E  ^91^RHRGV^95^ (11) --- ^107^CIQVL^111^(12)^124^GT^125^ ---  2 ^115^LGLIL^119^(11) --- ^131^CGPLW^135^(11)^147^GV^148^ ---  M  ^89^LGLSL^93^ (11) --- ^105^CGPTV^109^(11)^121^GL^122^ ---  X  ^92^LGLSL^96^ (11) --- ^108^CGPTV^112^(11)^124^GL^125^ ---  L  ^95^LGMTL^99^ (11) --- ^111^CDPGL^115^(11)^127^GL^128^ ---  10 ^106^MHLGM^110^(13) --- ^124^CAPLW^128^(11)^140^GI^141^ --- |

Table 1. (Continues)

|  | A0-A4 Ca^2+^ B0-B4 C1-C2 Ca^2+^  (Strand 1) (Strand 2) (Strand 3) |
| --- | --- |
| “Cage”  “FG-GAP” | -φφGφ-  -P- -G-  -FG- -GAP- |
| Blade 3  (without  the  I-domain) | IIb ^167^CEAGF^171^(12) --- ^184^GAPGG^188^(4) ^193^GL^194^ ---  V ^155^CQGGF^159^(12) --- ^172^GGPGS^176^(4) ^181^GQ^182^ ---  5 ^205^CQGGF^209^(12) --- ^222^GGPGS^226^(4) ^231^GQ^232^ ---  8 ^200^CQAGF^204^(12) --- ^217^GGPGS^221^(4) ^226^GQ^227^ ---  4 ^198^CQAGI^202^(11) --- ^214^GAPGS^218^(4) ^223^GS^224^ ---  3 ^197^CQLGT^201^(11) --- ^213^GAPGA^217^(4) ^222^GN^223^ ---  7 ^197^CQQGT^201^(13) --- ^215^GAPGT^219^(4) ^224^GL^225^ ---  6 ^188^CQQGV^192^(13) --- ^206^GAPGT^210^(4) ^215^GI^216^ ---  9 ^159^CQAGI^163^(11) --- ^175^GAPGS^179^(4) ^184^GT^185^ --- |
| Blade 3  (with the  I-domain) | 11 ^367^SQTGF^371^(11) --- ^383^GAVGA^387^(4) ^392^GA^393^ ---  D ^351^SQEGF^355^(11) --- ^367^GAVGS^371^(5) ^377^GA^378^ ---  1 ^378^SQTGF^382^(11) --- ^394^GAVGA^398^(4) ^403^GT^404^ ---  E ^402^AQIGF^406^(12) --- ^419^GAVGA^423^(4) ^428^GG^429^ ---  2 ^379^SQVGF^383^(13) --- ^397^GAVGA^401^(4) ^406^GT^407^ ---  M ^351^SQEGF^355^(11) --- ^367^STVGS^371^(4) ^376^GG^377^ ---  X ^352^AQEGF^356^(11) --- ^368^GAVGS^372^(4) ^377^GG^378^ ---  L ^350^SSSGI^354^(11) --- ^366^GAVGA^370^(4) ^375^GG^376^ ---  10 ^373^SQIGF^377^(11) --- ^389^GMVGA^393^(3) ^397^GG^398^ --- |
| Blade 4  (without  the  I-domain) | IIb ^233^GYWGY^237^(5)^243^EFDGD[4]E^252^(3)^256^GAPTW^260^(4) ^265^GA^266^(5)^272^S  V ^220^SYLGY^224^(5)^230^DFNGDGIDD^238^(3)^242^GVPRA^246^(4) ^251^GM^252^(5)^258^G  5 ^270^SYLGY^274^(5)^280^EFSGDDTED^288^(3)^292^GVPKG^296^(4) ^301^GY^302^(5)^308^G  8 ^265^SYLGY^269^(5)^275^EFTGDSQQE^283^(3)^287^GIPRG^291^(4) ^296^GY^297^(5)^303^S  4 ^249^SYLGY^253^(17) --- ^271^GAPQH^275^(3) ^279^GK^280^ ---  3 ^248^LYIGY^252^(18) --- ^271^GAPRH^275^(3) ^279^GA^280^ ---  7 ^261^SYLGF^265^(18) --- ^284^GAPRA^288^(3) ^292^GA^293^ ---  6 ^256^SYLGF^260^(18) --- ^279^GAPRA^283^(3) ^287^GA^288^ ---  9 ^245^TYLGY^249^(17) --- ^267^GAPQD^271^(3) ^275^GK^276^ --- |
| Blade 4  (with the  I-domain) | 11 ^423^AYLGY^427^(16) --- ^444^GAPRF^448^(3) ^452^GK^453^ ---  D ^402^SYLGY^406^(15) --- ^422^GAPRY^426^(3) ^430^GK^431^ ---  1 ^434^SYLGY^438^(16) --- ^455^GQPRY^459^(3) ^463^GQ^464^ ---  E ^459^SYLGY^463^(15) --- ^479^GAPQY^483^(3) ^487^GA^488^ ---  2 ^435^SYLGY^439^(15) --- ^455^GAPRA^459^(3) ^463^GQ^464^ ---  M ^402^AYLGY^406^(15) --- ^422^GAPRY^426^(3) ^430^GL^431^ ---  X ^403^SYLGY^407^(15) --- ^423^GAPRY^427^(3) ^431^GK^432^ ---  L ^402^GYLGY^406^(16) --- ^423^GAPRY^427^(3) ^431^GR^432^ ---  10 ^429^AYLGY^433^(16) --- ^450^GAPRF^454^(3) ^458^GK^459^ --- |
| Blade 5  (without  the  I-domain) | IIb ^287^SYFGH^291^(5)^297^DVNGDGRHD^305^(3)^309^GAPLY^313^(12)^326^GR^327^(5)^333^Q  V ^274^AYFGF^278^(5)^284^DINGDDYAD^292^(3)^296^GAPLF^300^(12)^313^GQ^314^(5)^320^Q  5 ^324^SYFGY^328^(5)^334^DVNGDGLDD^342^(3)^346^GAPLL^350^(12)^363^GR^364^(5)^370^Q  8 ^319^SYFGY^323^(5)^329^DVNSDGLDD^337^(3)^341^GAPLF^345^(12)^358^GQ^359^(5)^365^Q  4 ^304^SYFGA^308^(5)^314^DLNADGFSD^322^(3)^326^GAPMQ^330^(6) ^337^GR^338^(5)^344^N  3 ^305^AYFGS^309^(5)^315^DLNNDGWQD^323^(3)^327^GAPYY^331^(8) ^340^GA^341^(5)^347^N  7 ^318^SGFGY^322^(5)^328^DLNSDGWPD^336^(3)^340^GAPYF^344^(8) ^353^GA^354^(5)^360^N  6 ^314^SSFGY^318^(5)^324^DLNKDGWQD^332^(3)^336^GAPQY^340^(8) ^349^GA^350^(5)^356^N  9 ^302^SYFGS^306^(5)^312^DLNGDGLSD^320^(3)^324^GAPMF^328^(6) ^335^GQ^336^(5)^342^N |

Table 1. (Continues)

|  | A0-A4 Ca^2+^ B0-B4 C1-C2 Ca^2+^  (Strand 1) (Strand 2) (Strand 3) |
| --- | --- |
| “Cage”  “FG-GAP” | -φφGφ-  -P- -G-  -FG- -GAP- |
| Blade 5  (with the  I-domain) | 11 ^478^SYFGS^482^(5)^488^DIDGDGVTD^496^(4)^501^GAPMY^505^(7) ^513^GK^514^(5)^520^L  D ^455^SYFGA^459^(5)^465^DVDSDGSTD^473^(4)^478^GAPHY^482^(6) ^489^GQ^490^(5)^496^L  1 ^488^SYFGS^492^(5)^498^DIDKDSNTD^506^(4)^511^GAPMY^515^(8) ^524^GK^525^(5)^531^L  E ^512^SYFGS^516^(5)^522^DIDMDGSTD^530^(4)^535^AAPFY^539^(6) ^546^GR^547^(5)^553^L  2 ^489^SYFGS^493^(5)^499^DVDKDTITD^507^(4)^512^GAPMY^516^(8) ^525^GR^526^(5)^532^I  M ^455^AYFGA^459^(5)^465^DVDSNGSTD^473^(4)^478^GAPHY^482^(6) ^489^GQ^490^(5)^496^L  X ^456^SYFGA^460^(5)^466^DVDSDGSTD^474^(4)^479^GAPHY^483^(6) ^490^GQ^491^(5)^497^L  L ^458^SYFGG^462^(5)^468^DVDQDGETE^476^(4)^481^GAPLF^485^(6) ^492^GR^493^(5)^499^R  10 ^484^SYFGS^488^(5)^494^DTDRDGTTD^502^(4)^507^AAPMF^511^(8) ^520^GR^521^(5)^527^V |
| Blade 6  (without the  I-domain) | IIb ^354^GRFGS^358^(6)^365^DLDRDGYND^373^(3)^377^AAPYG^381^(5) ^387^GQ^388^(5)^394^G  V ^338^ARFGS^342^(6)^349^DLDQDGFND^357^(3)^361^AAPYG^365^(5) ^371^GI^372^(5)^378^G  5 ^390^GRFGS^394^(6)^401^DLDQDGYND^409^(3)^413^GAPFG^417^(5) ^423^GV^424^(5)^430^G  8 ^384^GRFGS^388^(6)^395^DLNQDGYND^403^(3)^407^GVPFA^411^(5) ^417^GK^418^(5)^424^G  4 ^366^ARFGE^370^(6)^377^DIDNDGFED^385^(3)^389^GAPQE^393^(4) ^398^GA^399^(5)^405^G  3 ^367^SAFGL^371^(6)^378^DINQDGFQD^386^(3)^390^GAPFE^394^(2) ^397^GK^398^(5)^404^S  7 ^379^SMFGI^383^(6)^390^DLNQDGFPD^398^(3)^402^GAPFD^406^(2) ^409^GK^410^(5)^416^G  6 ^375^SMFGI^379^(6)^386^DINQDGYPD^394^(3)^398^GAPYD^402^(2) ^405^GK^406^(5)^412^G  9 ^362^AHFGE^366^(6)^373^DLDNDGFPD^381^(3)^385^GAPKE^389^(4) ^394^GA^395^(5)^401^G |
| Blade 6  (with the  I-domain) | 11 ^540^ARFGS^544^(6)^551^DLNQDSYND^559^(3)^563^GAPLE^567^(4) ^572^GA^573^(5)^579^G  D ^519^GRFGA^523^(6)^530^DVNEDKLID^538^(3)^542^GAPGE^546^(4) ^551^GA^552^(5)^558^G  1 ^569^ARFGT^573^(6)^580^DLNLDGFND^588^(3)^592^GAPLE^596^(4) ^601^GA^602^(5)^608^G  E ^575^ARFGF^579^(6)^586^DLSQDKLTD^594^(3)^598^GAPLE^602^(2) ^605^GA^606^(5)^620^G  2 ^552^TRFGS^556^(6)^563^DINMDGFND^571^(3)^575^GSPLE^579^(4) ^584^GA^585^(5)^591^G  M ^519^GRFGA^523^(6)^530^DVNGDKLTD^538^(3)^542^GAPGE^546^(4) ^551^GA^552^(5)^558^G  X ^519^GRFGA^523^(6)^530^DVNGDKLTD^538^(3)^542^GAPGE^546^(4) ^551^GA^552^(5)^558^G  L ^519^GRFGE^523^(6)^530^DINGDGLVD^538^(3)^542^GAPLE^546^(2) ^549^GA^550^(5)^556^G  10 ^547^ARFGF^551^(6)^558^DLNQDGFAD^566^(3)^570^GAPLE^574^(4) ^579^GA^580^(5)^586^G |
| Blade 7  (without the  I-domain) | IIb ^415^SAFGF^419^(6)^426^DIDDNGYPD^434^(3)^438^GAYGA^442^(0) ^443^NQ^444^(5)^450^A  V ^402^PSFGY^406^(6)^413^DIDKNGYPD^421^(3)^425^GAFGV^429^(0) ^430^DR^431^(5)^437^A  5 ^454^DFFGS^458^(6)^465^DLDGNGYPD^473^(3)^477^GSFGV^481^(0) ^482^DK^483^(5)^489^G  8 ^448^SGFGF^452^(6)^459^DIDKNDYPD^467^(3)^471^GAFGT^475^(0) ^476^GK^477^(5)^483^A  4 ^428^SMFGQ^432^(6)^439^DADNNGYVD^447^(3)^451^GAFRS^455^(0) ^456^DS^457^(5)^463^T  3 ^428^ATFGY^432^(6)^439^DVDENFYPD^447^(3)^451^GSLS-^454^(0) ^455^DH^456^(5)^462^A  7 ^437^KSFGY^441^(6)^448^DMDGNQYPD^456^(3)^460^GSMA-^463^(0) ^464^DT^465^(5)^471^A  6 ^430^PYFGY^434^(6)^441^DLDRNSYPD^449^(3)^453^GSLS-^456^(0) ^457^DS^458^(5)^464^S  9 ^424^RMFGQ^428^(6)^435^DMDGNGYPD^443^(3)^447^GAFMS^451^(0) ^452^DS^453^(5)^459^A |
| Blade 7  (with the  I-domain) | 11 ^602^QYFGC^606^(6)^613^DLNEDGLID^621^(3)^625^GALG-^628^(0) ^629^NA^630^(5)^636^R  D ^582^QYFGQ^586^(6)^593^DLTQDGLMD^601^(3)^605^GARG-^608^(0) -Q^609^(5)^615^S  1 ^631^KFFGQ^635^(6)^642^DLNGDGLTD^650^(3)^654^GGLG-^657^(0) ^658^GA^659^(5)^665^R  E ^643^QYFGM^647^(6)^654^DISGDGLAD^662^(3)^666^GTLG-^669^(0) -Q^670^(5)^676^S  2 ^616^QYFGR^620^(6)^627^DLNGDSITD^635^(3)^639^GAFG-^642^(0) -Q^643^(5)^649^S  M ^582^QYFGQ^586^(6)^593^DLTMDGLVD^601^(3)^605^GAQG-^608^(0) -H^609^(5)^615^S  X ^582^QYFGQ^586^(6)^593^DLTQDGLVD^601^(3)^605^GARG-^608^(0) -Q^609^(5)^615^T  L ^579^QWFGR^583^(6)^590^DLEGDGLAD^598^(3)^602^GAES-^605^(0) -Q^606^(5)^612^S  10 ^609^SYFGR^613^(6)^620^DLDGDDLVD^628^(3)^632^GAQG-^635^(0) -A^636^(5)^642^S |
